# Polymorphism of the melatonin receptor 1A (*MNTR1A*) gene and association with seasonality of reproductive activity in a local Greek sheep breed

**DOI:** 10.1186/s40709-016-0050-y

**Published:** 2016-04-29

**Authors:** Ioannis A. Giantsis, George P. Laliotis, Olympia Stoupa, Melpomeni Avdi

**Affiliations:** Department of Animal Production, Faculty of Agriculture, Aristotle University of Thessaloniki, Thessaloniki, Greece

**Keywords:** Melatonin, RFLPs, Sheep, Reproduction, Seasonality, *MNTR1A* gene

## Abstract

**Background:**

Sheep’s reproductive physiology in temperate latitudes (such as Greece), is characterized by seasonality and is also regulated by photoperiodic exposure. Melatonin is the key hormone involved in this regulation. However, the melatonin secretion and therefore the ewes reproductive activity underlies variation, proposed to be linked with the melatonin receptor subtype 1A (*MNTR1A*) gene structure. This study was designed to investigate the polymorphism of the *MNTR1A* gene in a local Greek sheep breed and to determine its potential association with reproductive seasonality.

**Results:**

Two groups of farmed ewes, each consisted of 30 individuals, were chosen. Males were introduced in both groups in spring (April). The first group consisted of ewes that showed reproductive activity in spring (May), while the second of ewes that showed reproductive activity 3 months later, in summer. The PCR–RFLP methodology was carried out on a 824-bp DNA fragment of the *MTNR1A* exon 2 using the *Rsa*I restriction endonuclease. The electrophoretic procedure revealed three genotypes, *C/C*, *C/T* and *T/T*. Specifically, 44 animals showed the *C/C* genotype (28 from the first group and 16 from the second), 14 the *C/T* genotype (2 from the first and 12 from the second) and 2 animals had the *T/T* genotype (both from the second group).

**Conclusions:**

Statistical analysis indicated a positive correlation between genotype and reproductive seasonality, with *C/C* genotype playing a crucial role in out-of-season reproduction activity.

## Background

Reproductive activity in sheep living in temperate latitudes (such as Greece), exhibits a seasonal variation that it is regulated by photoperiodic exposure. This trait, known as reproductive seasonality, results in concentration of births in certain periods of the year. More specifically, ewes show high reproductive efficiency the period when the dark hours of the day begin to increase, namely from summer until winter [1, 2]. Nevertheless, a big variety of commercially important or traditionally made Greek cheeses, such as “feta”, “graviera” and “kefalotyri”, are produced by ovine milk, which, hence, remains in demand by the market throughout the whole year. Similarly, lamb meat is also in demand throughout the year. Despite manipulations that have been developed, such as the male induced ovulation (male effect), hormonal treatment and general domestication practices that mitigate the photoperiodism, there still exist anestrus periods in sheep [2]. Therefore, the finding of less sensitive to reproductive seasonality genotypes, would be a fair alternative in order to rear polyestrus animals that could show reproductive activity regardless of the period of the year, avoiding that way any hormonal treatment.

The chemical signal for photoperiodism in mammals is considered to be the hormone melatonin, playing a key role in the phenomenon of the reproduction seasonality [3, 4]. Melatonin is synthesized by the pineal gland and secreted in high concentration during the dark hours of the day, influencing positively the reproduction when the night lasts longer. However, the melatonin secretion and consequently the reproductive seasonality underlies variation in the different sheep breeds, e.g. Texel and Serres breeds have long anestrus seasons, while Merinos, Romanov and Chios breeds are characterized by lower seasonality [5], and the Chinese Small Tail Han and Hu sheep breeds display entirely non seasonal reproductive physiology [6]. Two specific receptors are involved in the melatonin secretion, of which, only the melatonin receptor subtype 1A (*MTNR1A*) is considered to be a candidate gene that mediates the photoperiodic reproductive seasonality in sheep [4, 7]. Particularly, the structure and polymorphism of exon 2 of the *MTNR1A* has been investigated in several sheep breeds [2, 7–9] and specific genotypes have been associated with out-of-season reproductive activity [4, 6, 10]. Although ovine milk is a very important product for Greece and the raw material for many products of protected designation of origin (regulation: 2081/92/EEC), the *MTNR1A* gene polymorphism has not been examined in any sheep breed in Greece. The present work is the first effort to study the *MTNR1A* exon 2 polymorphism in a local Greek sheep breed and its potential association with reproductive seasonality.

## Results

**Fig. 1:**
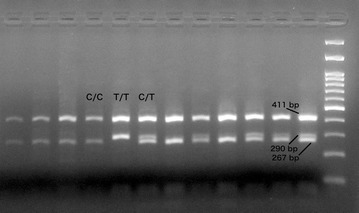
Sheep genotypes in agarose gel. Representative electrophoretic profiles of the observed genotypes after the digestion of MNTR1A gene with *Rsa*l restriction endonuclease. Marker: GeneRuler 100 bp DNA ladder

The 824-bp coding sequence of the *MTNR1A* was successfully amplified in all 60 ewe samples, providing a clear band without non specific products. *Rsa*I restriction enzyme recognizes and cuts the sequence GT^AC. Digestion with *Rsa*I revealed four cleavage sites, one of which was polymorphic, corresponding to the position 606 of the reference sequence U14109 [11]; when there was a C in position 606 (sequence positions 603–606: GTAC) the enzyme recognized the cleavage site, while when there was a T in position 606 (603–606: GTAT) the enzyme did not recognize any cleavage site. Hence, two fragments, 290 and 411 bp in length, were observed in the agarose gel when the polymorphic site was present, whereas fragments 267 and 411 bp in length were observed when it was absent (Fig. 1). The remaining cleavage sites produce fragments of 53, 23 and 70 bp [6]. Eventually, the presence and the absence of the polymorphic cleavage site defined two alleles, named by the nucleotides, *C* and *T*, respectively. All possible combinations of the two alleles were observed, revealing three genotypes namely *C/C*, *C/T* and *T/T* (Fig. 1; Table 1). Forty-four animals showed the *C/C* genotype (28 from the group A and 16 from the group B), 14 the *C/T* genotype (2 from the group A and 12 from the group B) and 2 animals had the *T/T* genotype (both from the group B). Allele and genotype frequencies are shown in Table 1. The difference of the genotype frequencies between the two groups was statistically significant (*χ*^2^ = 12.416, df = 2, *p* = 0.002). The population was found to deviate from Hardy–Weinberg equilibrium (*p* = 0.51), probably due to the low heterozygote frequency. The allele *C* and the genotype *C/C* frequencies were extremely high (Table 1) in the group A (animals that showed an early occurrence of reproductive performance), while the *T/T* genotype was observed only in the group B (animals that showed a delayed occurrence of reproductive performance), suggesting a correlation between genotype and seasonality in regard to the occurrence of reproductive activity. Statistical analysis confirmed this positive correlation between the two genotypes (*C/T* and *T/T*) and seasonal reproductive activity (r = 0.44, *p* < 0.01).

**Table 1:** Alleles and genotype absolute frequencies of the PCR–RFLP analysis in the Greek sheep breed

	Allele frequency		Genotype frequency		
	*C*	*T*	*C/C*	*C/T*	*T/T*
Group A	0.97	0.03	0.93 (28)	0.07 (2)	0
Group B	0.73	0.27	0.53 (16)	0.4 (12)	0.07 (2)
All samples	0.85	0.15	0.73 (44)	0.23 (14)	0.03 (2)

The values in the brackets represent the number of individuals showing each genotype

## Discussion

A lot of economically important Greek cheeses are produced by ovine milk keeping it in consistently high demand throughout the year. However, reproductive activity in sheep reared in Greece, is characterized by photoperiodism and reproductive seasonality resulting in concentrations of births and milk production in specific periods of the year. These phenomena constitute significant limitations for farmers. Although hormonal treatment eliminates the impact of this reproductive trend by causing more estrus, it does not reflect the consumers’ constant demand for free-hormone products as well as its running costs are considered unaffordable.

Several genetic analyses in various sheep breeds have shown an association of particular genotypes of the exon 2 of the *MTNR1A* gene with expression of reproductive seasonality [4, 6, 9, 10, 12]. In these studies, the *C/C* genotype exhibited the highest frequency and was linked to non seasonal estrus, although the point mutation of allele *T* is silent [12], resulting in tyrosine in both cases (both allele *C* and *T*).

In the present study, we analyzed the structure of the *MTNR1A* gene for the first time in a local Greek sheep breed. The allele *C* was the most frequent allele in the examined population while the distribution of the *C/C* genotype was extremely high (0.93, Table 1) in the ewes that responded immediately to the male effect (group A). The latter is in accordance with the levels reported on non seasonal breeds or intermittently polyestrous breeds such as the Indian Magra and Garole breeds or the Small Tail Han and Hu breeds [6, 8]. On the contrary, the *T/T* genotypes were only found in ewes that showed estrus in middle summer, while the *C/C* and the *C/T* genotypes in this group were found in lower levels, similarly to the Indian Malpura and Avikalin sheep breeds [8] or the Suffolk, the Dorset and the German Merino breeds [6]. Thus, in accordance with the Sarda sheep breed [4], in the present study, groups of animals of the same breed but with different response to reproductive activity, revealed different allelic and genotype frequencies of the *MTNR1A* gene. Interestingly, another native Eastern Mediterranean breed, Chios sheep breed, did not show any polymorphism of the *MTNR1A* gene at the same locus [13], probably due to the fact that this breed is generally not characterized by intense seasonality [5].

In conclusion, particular genotypes seem to have an additive effect in sheep’s fertility in regard to out-of-season reproduction activity, with allele *C*, and genotype *C/C* frequently present in ewes that show a non delay in the occurrence of reproductive activity, while the allele T seems to have a non beneficial impact on that activity. Therefore, in order to reduce seasonality in sheep reared in countries where ovine milk is an economically important product that remains in demand during the entire year, marker assisted selection programs using PCR–RFLP analysis of the *MTNR1A* gene, could be a valuable strategy. However, before designing a particular selection scheme, more knowledge is needed about the mechanisms underlying the function of the different alleles of the *MTNR1A* gene.

## Methods

Two groups of ewes (namely hereafter group A and group B), originating from a Greek local breed of Western Greece, were chosen for the present study. Males were introduced in both groups in spring (middle April). Each group consisted of thirty (30) ewes; group A was made up by ewes that showed an early reproductive activity (middle May), while the group B by ewes that showed a 3 month delay in the occurrence of reproductive activity, thus in middle summer (early August). All the animals were in good health condition, were fed with the identical diet and were hormone untreated. Blood samples were taken from the jugular vein of each one of the 60 animals and were collected in 10 ml tubes containing sodium heparin as an anticoagulant additive (BD Vacutainer Systems, Plymouth, UK). Total DNA was extracted using the Nucleospin blood kit (Macherey–Nagel, Germany) according to the manufacturer’s protocol. A 824-bp DNA segment of the exon 2 of the *MTNR1A* was amplified with PCR using the primers and the protocol described by Messer et al. [14]. Restriction fragment length polymorphism (RFLP) analysis was performed on the amplified products, using the *Rsa*I restriction endonuclease. Eight microliters of each PCR product was digested for 3 h at 37 °C, with 8 units of *Rsa*I (Thermo Scientific, Dreieich, Germany), in a reaction of 20 μl total volume, containing 2 μl of 10× buffer Tango and 9.2 μl nuclease-free water. Restriction patterns were visualized in a 2.5 % agarose gel, stained with ethidium bromide and the fragments were sized according to a 100 bp DNA ladder (GeneRuler 100 bp, Fermentas, Vilnius, Lithuania). Statistical analysis was carried out using the SPSS package (ver. 13.0). Pearson correlation was used in order to examine the association of the *MTNR1A* genotypes with reproduction seasonality, while a *χ*^*2*^ test was performed to determine any deviation of the population from Hardy–Weinberg equilibrium and to evaluate the differences between the observed genotype frequencies in the two groups.

All animal manipulations were carried out according the EU Directive on the protection of animals usage for scientific purposes (2010/63/EU). No other specific permits were required since the sample collection did not involve endangered or protected species.
